# Modulation of Dietary Fatty Acids in an Open-Label Study Improves Psoriasis and Dampens the Inflammatory Activation Status

**DOI:** 10.3390/nu15071698

**Published:** 2023-03-30

**Authors:** Anja Saalbach, Anna-Theresa Seitz, Johannes Kohlmann, Lena Kalweit, Lisa Vogt, Lars Selig, Kathrin M. Engel, Jan C. Simon

**Affiliations:** 1 Department of Dermatology, Venerology and Allergology, Faculty of Medicine, Leipzig University, Philipp Rosenthal Str. 23, 04103 Leipzig, Germany; anna-theresa.seitz@medizin.uni-leipzig.de (A.-T.S.); lena.kalweit@medizin.uni-leipzig.de (L.K.); lisa.vogt@medizin.uni-leipzig.de (L.V.); jan-christoph.simon@medizin.uni-leipzig.de (J.C.S.); 2 Department of Medicine, Division of Nutritional Medicine, Faculty of Medicine, Leipzig University, 04103 Leipzig, Germany; lars.selig@medizin.uni-leipzig.de; 3 Institute of Medical Physics and Biophysics, Faculty of Medicine, Leipzig University, 04107 Leipzig, Germany; kathrin.engel@medizin.uni-leipzig.de

**Keywords:** nutrition, psoriasis, obesity, fatty acids, inflammation

## Abstract

Obesity and high abdominal fat mass are risk factors for developing the chronic inflammatory skin disease psoriasis. They are associated with increased incidence, prevalence and severity of the disease. A positive effect of weight loss on psoriasis activity has been shown in several studies. Obesity-related factors such as the dysregulation of glucose and lipid metabolism, the activation of adipose tissue and resultant persistent low-grade inflammation have been discussed as links of obesity and inflammatory diseases. Recently, we demonstrated a critical role of free fatty acids (FFAs) in obesity-mediated exacerbation of psoriatic skin inflammation in both mice and humans. In the present study, we translated these findings into a therapeutic intervention. An open-label study focusing on the dietary reduction of FFAs was conducted in patients with mild-to-moderate plaque psoriasis, and disease severity and serum markers of inflammation were analyzed. Here, we show that such a dietary intervention improves psoriatic disease activity independently of weight loss. Diet-related metabolic changes, such as a reduction in saturated free fatty acids (SFAs), may thus be more important than weight loss itself. Moreover, dietary intervention inhibited the overall pro-inflammatory activation status in patients, as shown by analysis of serum inflammatory parameters using the Olink platform. From our pilot study, we conclude that dietary intervention focusing on SFA reduction has the capacity to reduce disease activity and general inflammatory status in psoriasis patients.

## 1. Introduction

Obesity has reached epidemic proportions, affecting more than 650 million people worldwide [1]. It is associated with a reduced disease-free life and premature death and predisposes individuals to various diseases, including type 2 diabetes, cardiovascular diseases, chronic kidney disease, cancer and infections [1]. In addition, obesity increases the incidence and severity of many inflammatory/autoimmune disorders including hepatic steatosis, inflammatory bowel disease, colitis, atherosclerosis and psoriasis [2,3,4,5,6,7]. Consistent with human data, obesity exacerbates inflammation in several animal models of chronic inflammatory diseases such as psoriatic dermatitis, inflammatory bowel disease and colitis [4,5,6,8,9,10,11,12].

Epidemiological studies demonstrate a substantial impact of obesity on the incidence and severity of psoriasis. For example, obesity and high abdominal fat mass double the risk for developing psoriasis [13,14]. Obesity-related factors such as disturbed glucose and lipid metabolism, the activation of adipose tissue due to the excessive fat deposition and the resulting increased levels of pro-inflammatory adipokines, cytokines and chemokines have been discussed as links between obesity and inflammation [15,16]. Recently, we demonstrated a critical involvement of free fatty acids (FFAs) in obesity-mediated exacerbation of psoriatic skin inflammation [17,18]. Among the obesity-related factors, only serum FFA levels correlated with the severity of skin inflammation in psoriasis patients. In a mouse model, we proved the central role of FFAs in exacerbating psoriasis-like skin inflammation. Non-obese/non-diabetic mice with elevated FFA levels developed a significantly more severe psoriasis-like skin inflammation, which could be prevented by the dietary reduction of FFA [18]. Mechanistically, we showed that increased concentrations of saturated free fatty acids (SFAs), together with the danger molecule S100A9, stimulate inflammasome activation in macrophages, leading to an increased IL-1b secretion, which propagates S100A9 production in keratinocytes. S100A9, in turn, attenuates the differentiation of anti-inflammatory M2-like macrophages, which develop a pro-inflammatory activation profile, thereby initiating a vicious cycle of skin inflammation in obesity. Breaking the vicious cycle of S100A9 overexpression by a dietary reduction in FFAs restored M2-like macrophage activation and ameliorated skin inflammation [19]. Taken together, dietary FFAs seem to play an essential role in the obesity-mediated amplification of psoriatic skin inflammation.

In the present study, we translated these findings into a therapeutic approach. In an open-label study, we conducted a dietary intervention in patients with mild-to-moderate plaque psoriasis and analyzed disease severity and serum inflammatory markers. Indeed, a dietary intervention focused on reducing SFAs improved disease severity and decreased a wide range of serum inflammatory biomarkers detected by the Olink Proteomics Platform.

## 2. Methods

### 2.1. Human Study

We included patients with mainly mild to moderate psoriasis vulgaris aged ≥18 who were treated for psoriasis at our medical center (Supplementary Table S1). Three patients had severe psoriasis but declined any systemic treatment. All systemic and topical treatments were permitted but could not be changed at least 4 weeks before and during dietary intervention; otherwise, patients were excluded. The study was approved by the local ethics committee (#163/18-ek), and all patients gave written informed consent. Patients were randomized into two groups. Group 1 (n = 16) started directly with a 12-week dietary intervention. Group 2 (n = 17) kept their usual diet for three months before they started with the same 12-week dietary intervention as patients of group 1. Dietary intervention was designed with the aim of reducing the uptake of SFA (Supplementary Table S2). In detail, the meals were initially replaced by a formula diet according to the manufacturer’s instructions and subsequently based on a modified Mediterranean diet. Specifically, in the first week, all meals were replaced by one portion of the formula diet. A total of 100 g formula diet powder were dissolved in up to 300 mL of still water (or 200 mL of low-fat milk or 200 mL of buttermilk or sugar-free tea), and 2 teaspoons (=6 g) vegetable oil (canola oil, soybean oil, linseed oil, walnut oil) were added. If needed, sugar-free additives (cinnamon, turmeric, baking cocoa) could be added. Patients were asked to take the formula diet in the correct dosage according to the plan, even on vacation, holidays or birthdays. In the second and third week, only two meals were replaced by the formula diet. The third meal consisted of components of the Mediterranean diet, for example, 1–2 slices of whole-meal bread, fat-reduced margarine and a choice of fat-reduced cheese or fat-reduced sausage or ham. In the fourth week, one meal was replaced by the formula diet. The other two meals could be freely chosen by the patients according to Mediterranean referral. The Mediterranean diet was modified according to the recommendations for a wholesome diet of the German Nutrition Society (DGE, https://www.dge.de/ernaehrungspraxis/vollwertige-ernaehrung/10-regeln-der-dge/en/ (accessed on 22 January 2023)) with the additional recommendation of a low SFA intake (Supplementary Table S2). Throughout the study, patients were accompanied by professional nutrition specialists. In the course of the intervention period, a total of 8 nutrition consultations took place. Clinical data including Psoriasis Area and Severity Index (PASI) and body mass index (BMI) were recorded 12, 10, 8 and 4 weeks before start of the intervention, at the start of the intervention and 2, 4, 8 and 12 weeks after the intervention. Serum was collected at each visit.

### 2.2. Serum Biomarkers

Inflammatory serum biomarkers were detected by the Olink Proteomics Platform (Olink Proteomics, Uppsala, Sweden) using the human Olink target 96 inflammation panel. Analysis was performed by Olink.

### 2.3. Lipid Extraction and Analysis of Free Fatty Acids

Serum aliquots (60 µL) were mixed with 1 mL methanol and centrifuged at 13,000× *g* for 2 min to precipitate proteins. Lipids were extracted from the supernatant by the addition of 1 mL chloroform and water according to Bligh and Dyer [20]. After vigorous shaking, samples were centrifuged for phase separation at 3000 rpm for 5 min. The lower (organic) phase was withdrawn, and lipid extraction was repeated by the addition of chloroform, vigorous shaking and centrifugation. Both organic phases of a sample were combined, evaporated to dryness and re-dissolved in 200 µL chloroform. Ten µL of the organic extracts were separated by high-performance thin-layer chromatography (HPTLC). The lipids in the free fatty acid (FFA) spot were automatically eluted by a Plate Express™ TLC plate reader (Advion, Ithaca, NY, USA) with methanol as a solvent and directly analyzed by electrospray ionization mass spectrometry (ESI MS) as described [21]. Total intensities of FFA and, from these, ratios for each FFA were calculated. Total FFA concentration was detected in serum samples by the Free Fatty Acid Quantitation Kit (Sigma-Aldrich; without lipid extraction) according to the manufacturer’s protocol and detected via UV-spectrometer measurement (Hitachi U-2000, Hitachi Medical Systems America, Inc., Twinsburg, OH, USA) or Synergy HT (BioTek). Specific FFA concentrations were calculated from the percentage of specific FFA and total FFA.

### 2.4. Statistics

For statistical correlation analyses, IBM SPSS Statistics software version 27.0 (IBM, Armonk, NY, USA) was employed. The non-parametric Spearman’s rank correlation test was used to assess univariate relationships between clinical and biochemical markers. A multivariate regression analysis was performed for specific mediators adjusted for BMI, waist–hip ratio (WHR) and serum triacylglycerol concentration (TAG). Non-normally distributed variables as assessed by the Shapiro–Wilk test were logarithmically transformed prior to multivariate testing. Values of *p* < 0.05 were considered to be significant. The different degrees of significance were indicated by asterisks (* *p* < 0.05; ** *p* < 0.01; *** *p* < 0.001). Data presentation was performed using GraphPad 9.5.

## 3. Results

### 3.1. Dietary Modulation of FFA Reduces Disease Severity in Patients with Psoriasis

**Figure 1:**
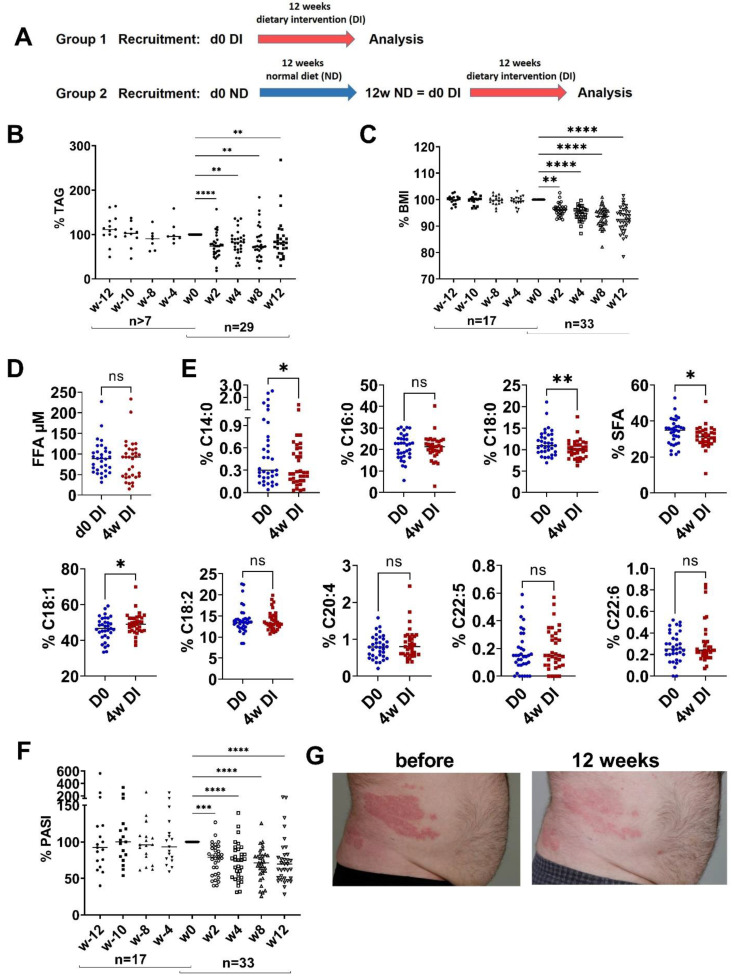
Dietary intervention reduces TAG, BMI and PASI. (**A**) Patients with mild-to-moderate psoriasis were randomized into two groups: group 1 with dietary intervention (DI) for 12 weeks (w); group 2 with normal diet (ND) for 12 weeks followed by a change to DI for 12 weeks. (**B**,**C**) Percent reduction in serum triglycerides (TAG) and body mass index (BMI) before and after dietary intervention compared to w0 at indicated time points. Number of patients is indicated. (**D**) Total serum free fatty acid (FFA) before (d0 DI) and 4 weeks of DI (4w DI). (**E**) Percentage of specific FFA before and 4 weeks after DI. SFA is the sum of C14:0, C16:0 and C18:0. (**F**) Percent reduction in Psoriasis Area and Severity Index (PASI) before and after DI compared to w0 at indicated time point. Number of patients is indicated. (**G**) Representative image of skin from patients before and 12 weeks after DI. (**B**,**C**,**F**) ANOVA with multiple comparisons and non-parametric data were used (Friedman test); ** *p* < 0.01, *** *p* < 0.001, **** *p* < 0.0001, compared to w0. (**D**,**E**) Paired *t*-test. * *p* < 0.05, ** *p* < 0.01, ns—not significant.

Recent data proved an essential role of elevated FFAs in the obesity-mediated amplification of skin inflammation in a mouse model of psoriasis-like inflammation as well as in patients with psoriasis [18]. In the present study, we investigated whether these findings could be translated into a therapeutic approach. Therefore, 30 patients with mild-to-moderate psoriasis and three patients with severe psoriasis were included in a dietary intervention program. All patients kept their individual pharmacological psoriasis treatment (Supplementary Table S1) for a period of at least 4 weeks before and during the intervention period. Patients in group 1 started directly with a 12-week dietary intervention (Supplementary Table S2; Figure 1A). Patients in group 2 maintained their normal diet for 3 months before they crossed over to the dietary intervention (Figure 1A). The primary endpoint of the study was the impact of dietary intervention on the severity of psoriasis as measured by PASI.

First, the dietary intervention in group 1 significantly reduced both TAG and BMI, demonstrating the compliance of the patients to the diet (Figure 1B,C). In contrast, neither the amount of TAG nor the BMI had changed at the end of the 3-month period of normal diet in group 2 (Figure 1B,C). During dietary intervention, the total amount of FFA did not change (Figure 1D), but the relative amounts of SFAs such as myristic acid (C14:0) and stearic acid (C18:0) were reduced (Figure 1E). The relative amount of the monounsaturated FFA oleic acid (C18:1) increased, and the polyunsaturated FFAs such as linoleic acid (C18:2) and docosahexaenoic acid (C22:6, DHA) were not affected by the dietary intervention. Eicosapentaenoic acid (C20:5, EPA) was not detected. Consistently and most importantly, psoriasis improved significantly during the dietary intervention (Figure 1F,G). Underlining the importance of the dietary intervention, psoriasis was not improved in group 2 at the end of the 3 months of normal diet. Only after the crossover of group 2 to the dietary intervention, psoriasis also improved in these patients, excluding any bias due to the particular settings of the clinical study.

### 3.2. Impact of Dietary Intervention on Inflammatory Serum Cytokine Pattern

To elucidate the mechanisms by which the dietary intervention reduced disease activity in patients, we analyzed the serum cytokine expression pattern by the Olink Proteomics Platform. The focus was on 92 human serum mediators of inflammation, of which 72 markers were detectable in the patients’ sera. To analyze the impact of the dietary intervention on these biomarkers, we calculated the percentage of PASI reduction and the reduction in biomarkers after 4 and 8 weeks. Correlation analysis revealed that about one-third of the mediators were dependent on disease severity, BMI or TAG (Table 1).

Improvement of psoriasis (determined as a decrease in the disease severity index PASI) correlated with the change in 13 biomarkers during the observation period. Univariate correlation analysis showed a positive correlation of serum CD6, CST5, IL-18, IL-18R, ENRAGE and CASP8 reduction at week 4 and IL-7, TGFb, IL-6, IL-18, CCL19, IL-10RB, IL-18R1 and INFγ reduction at week 8 (Table 1). Multivariate linear regression analysis indicated that CD6, ENRAGE, CASP8, IL-7, IL-6, IL-18 and INFγ reduction were positively correlated to PASI reduction independent of WHR, BMI and TAG (Table 2). IL-10RB, IL18R and CST5 showed a slight association, and the correlations to CCL19 and LAP-TGFβ were lost.

**Figure 2:**
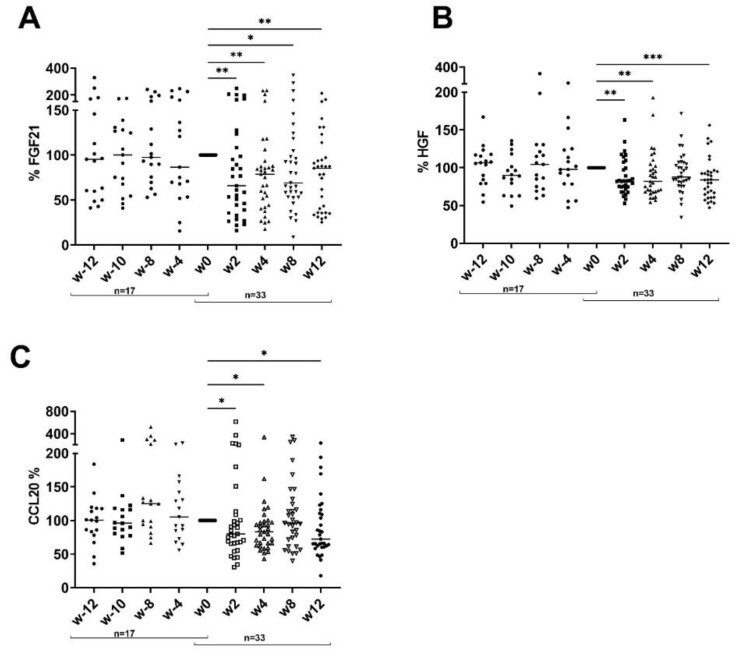
Dietary intervention reduces CCL20, FGF21 and HGF. Percent reduction in FGF21 (**A**), HGF (**B**) and CCL20 (**C**) before and after dietary intervention compared to w0 at indicated time point. Number of patients is indicated. ANOVA with multiple comparisons and non-parametric data were used (Friedman test). * *p* < 0.05, ** *p* < 0.01, *** *p* < 0.001 compared to w0.

In addition, correlation analysis revealed an association between 14 serum inflammatory markers and TAG reduction and between seven markers and BMI (Table 1). Moreover, CCL20, FGF21 and HGF were significantly reduced during the dietary intervention, although we did not observe any correlation with PASI, BMI or TAG (Figure 2).

Taken together, the dietary intervention affected a wide range of inflammatory markers, depending on the disease activity, BMI or TAG.

## 4. Discussion

Obesity is an independent risk factor for the incidence, prevalence and severity of psoriasis. Meta-analyses revealed that a higher BMI increases the risk of psoriasis onset. Moreover, obesity reduces treatment efficacy and may predict discontinuation of biological therapy [22]. Therefore, the Medical Board of the National Psoriasis Foundation recommends dietary weight reduction with a hypocaloric diet in overweight and obese patients with psoriasis. This recommendation is based on a meta-analysis that included 77,557 participants, 4534 of whom had psoriasis [23]. However, the underlying mechanisms are only poorly understood. Pro-inflammatory activation of adipose tissue resulting in increased adipokine, cytokine and chemokine secretions may link obesity and inflammation [15,16]. Insulin resistance and high glucose levels are known to promote inflammation. Recently, we have demonstrated a prominent role of FFA in obesity-mediated exacerbation of psoriatic skin inflammation [17,18]. We have shown that serum FFA levels correlate with the severity of skin inflammation in psoriasis patients. Moreover, a dietary reduction in SFAs improved psoriasis-like skin inflammation in the imiquimod-induced mouse model of psoriasis [17,18]. In the present study, this knowledge was translated into a dietary intervention approach in psoriatic patients. Patients with mainly mild to moderate plaque-type psoriasis were enrolled in a dietary intervention program focused on reducing dietary SFAs. The combination of a formula diet and a modified Mediterranean diet in our regime resulted in a significant reduction in serum TAG, proving the compliance of the patients and the success of the chosen diet with a focus on SFA reduction. In fact, our diet led to a significant decrease in serum SFA. In parallel, the patients’ BMI decreased significantly. However, total FFAs did not change during the dietary intervention. Serum FFA levels depend on many daily lifestyle factors that cannot be fully controlled in an outpatient setting. For example, chronic exercise reduces total FFA, whereas acute exercise increases it by inducing adipose tissue lipolysis. Chronic caloric restriction reduces FFAs, whereas acute fasting induces them. Stress, including beta-adrenergic stimulation, induces lipolysis and a subsequent increase in serum FFA. Smoking and sleeping habits, insulin resistance, obesity and dietary habits are other regulators of FFA release into the circulation [24].

Several studies have shown a positive effect of weight loss on psoriasis severity and treatment response. Diet and physical exercise may improve pre-existing psoriasis and prevent the de novo development of psoriasis [22]. Our dietary regime focusing on SFA reduction improved PASI by about 25% in patients who had stable disease activity under anti-psoriatic treatment. In our study, BMI reduction did not correlate with PASI reduction, suggesting that metabolic changes such as the SFA reduction might be more important than weight loss per se. Other metabolic factors such as short fatty acids, cholesterol or uric acid may also contribute to the beneficial effect of our diet on psoriasis severity.

We can exclude that study-related intensified medical care had an effect on the study outcome, since group 2, which did not receive any dietary intervention for the first three months, showed no change in TAG and BMI or PASI. Only after the crossover to the dietary intervention did metabolic parameters and disease severity improve in group 2. Thus, the dietary intervention alone is responsible for the observed effects.

In parallel, serum inflammatory biomarkers are down-regulated by dietary intervention. All of the regulated mediators, both the psoriasis-dependent and BMI/TAG-dependent markers, have been described previously to be related to psoriasis. Among the down-regulated markers, there are molecules known to mediate the interaction of antigen-presenting cells (APCs) and T cells (CD6, CD8a, CDCP1, SLAMF1), resulting in the activation of T cells and supporting a Th1 or Th17 response [25,26,27]. CD6 is expressed by lymphocytes and facilitates the interaction between T cells and APCs, resulting in the secretion of inflammatory mediators such as TNF-α, IFN-α and IL-6. CD6-deficient mice develop an attenuated psoriasis-like skin inflammation [25,28]. Based on this knowledge, inhibiting anti-CD6 antibodies were successfully used for the treatment of psoriasis. Dietary intervention reduces a wide variety of chemokines, which might attenuate the recruitment of inflammatory cells and break the vicious cycle of inflammation in psoriasis. For example, dietary intervention reduces CCL20, a major attractant of CCR6+ Th17 cells, the drivers of an IL-17A-rich cutaneous milieu. Signaling via the CCL20/CCR6 axis was shown to be more pronounced in the lesional skin of human psoriasis and in mouse models of psoriasis-like skin inflammation. Inhibition of the CCL20/CCR6 axis attenuated experimentally induced psoriasiform dermatitis. Thus, the CCL20/CCR6 axis could be a novel promising target for treating psoriasis [29]. Recently, a disulfide-linked CCL20 dimer was developed. This engineered dimer was able to bind and to activate CCR6 but inhibited T cell-mediated chemotaxis, resulting in a reduction in inflammation in an IL-23-induced mouse model of psoriasis [30]. In addition, pro-inflammatory and immune-modulatory cytokines or their receptors such as IL-18/IL-18R, IL-6, IL-7, IL-10/IL-10RB, TNF α and IFN-γ are decreased by dietary intervention. This is in accordance with a former study that showed a correlation between serum levels of IFN-γ, TNFα, IL-18 and IL-12 and psoriasis severity [31]. Additionally, IFN-γ was shown to inhibit the apoptosis of keratinocytes, promoting the hyper-proliferative state in psoriasis [31]. IL-7 is constitutively expressed by keratinocytes and dermal lymph vessels. Several studies demonstrated an increase in IL-7 in psoriasis. Moreover, blocking of IL-7Rα ameliorated imiquimod-induced inflammation in mice [32]. Another study showed that activated keratinocytes express IL-18 [33]. This chemokine is able to stimulate IFN-γ production by Th1 cells and IL-17 expression by Th17 lymphocytes, promoting a Th1/Th17 response [33]. Deletion of IL-18 reduces psoriasis-like skin inflammation in mice [34]. Many S100 proteins including S100A7/A8/A9/A12 are upregulated in psoriasis. Wilsman-Theis and colleagues reported that S100A12 (ENRAGE) shows the closest association with disease activity and therapeutic response [35]. For HGF, which was also reduced by dietary intervention, mitogenic, morphogenic and motogenic activities toward many epithelial cells were described [36]. In addition, HGF was shown to be an important angiogenic factor in the skin, acting indirectly by inducing VEGF expression in keratinocytes and directly by stimulating endothelial cell growth migration [37].

In summary, this study investigated the effect of an easy-to-use dietary intervention program focused on reducing saturated FFA on disease activity in psoriasis. It shows that such a dietary intervention alone has the capacity to reduce disease activity in treated patients by an additional 25%. This is independent of weight loss, suggesting that diet-related metabolic changes such as SFA reduction may be more important than weight loss itself. We emphasize that dietary intervention should not replace an anti-psoriatic treatment but rather represents a valuable add-on approach. It also affects a range of biomarkers, both psoriasis-specific cytokines and more general pro-inflammatory signals also found in other chronic (auto)immune diseases. Thus, dietary intervention with a reduced amount of SFA may be also helpful in other inflammatory autoimmune diseases in which an association of disease activity and obesity has been described.

## Figures and Tables

**Table 1:** Univariate correlations of inflammatory serum markers and disease activity, BMI and serum TAG.

		PASI w4	PASI w8	TAG w4	TAG w8	BMI w4	BMI w8
**PASI**	**r**			0.197	0.342	−0.059	0.169
	**p**			0.316	0.069	0.767	0.382
**WHR**	**r**	−0.021	−0.291	−0.128	0.087	0.089	0.144
	**p**	0.914	0.125	0.518	0.655	0.651	0.456
**BMI**	**r**	−0.059	0.169	0.375 *	0.460 *		
	**p**	0.767	0.382	**0.049**	**0.012**		
**TAG**	**r**	0.197	0.342			0.375 *	0.460 *
	**p**	0.316	0.069			**0.049**	**0.012**
**IL8**	**r**	0.187	0.215	0.226	0.192	−0.339	−0.280
	**p**	0.341	0.262	0.249	0.319	0.078	0.141
**VEGFA**	**r**	0.207	−0.100	0.262	0.149	−0.091	0.069
	**p**	0.290	0.604	0.178	0.440	0.646	0.723
**CD8A**	**r**	0.138	−0.011	0.317	0.167	−0.378 *	−0.103
	**p**	0.483	0.956	0.100	0.387	**0.047**	0.596
**MCP-3**	**r**	0.170	−0.091	0.170	−0.073	−0.405 *	−0.246
	**p**	0.386	0.640	0.386	0.705	0.032	0.198
**GDNF**	**r**	0.067	−0.125	−0.006	0.004	−0.116	0.168
	**p**	0.737	0.519	0.976	0.982	0.555	0.384
**CDCP1**	**r**	0.132	0.346	0.250	0.467 *	−0.234	0.180
	**p**	0.503	0.066	0.200	**0.011**	0.231	0.351
**CD244**	**r**	0.123	0.051	0.180	0.119	−0.318	−0.242
	**p**	0.534	0.792	0.360	0.538	0.100	0.205
**IL-7**	**r**	−0.008	0.429 *	0.300	0.219	−0.144	−0.107
	**p**	0.967	**0.020**	0.121	0.254	0.464	0.579
**OPG**	**r**	0.097	0.317	0.263	0.340	−0.012	−0.083
	**p**	0.622	0.094	0.177	0.071	0.953	0.668
**LAPTGF-beta-1**	**r**	0.201	0.400 *	0.097	0.341	0.055	0.133
	**p**	0.305	**0.032**	0.622	0.070	0.781	0.493
**uPA**	**r**	0.089	0.244	0.025	0.075	−0.348	−0.289
	**p**	0.652	0.202	0.901	0.698	0.070	0.128
**IL6**	**r**	0.118	0.561 **	0.054	0.243	−0.005	−0.172
	**p**	0.549	**0.002**	0.785	0.204	0.981	0.372
**IL-17C**	**r**	0.143	0.024	0.105	0.117	0.099	0.272
	**p**	0.468	0.901	0.597	0.546	0.616	0.154
**MCP-1**	**r**	0.234	0.046	0.223	0.154	−0.099	0.064
	**p**	0.232	0.813	0.253	0.426	0.616	0.741
**IL-17A**	**r**	0.120	0.152	0.232	0.264	0.087	0.281
	**p**	0.544	0.431	0.235	0.166	0.661	0.140
**CXCL11**	**r**	0.235	0.296	0.035	0.066	−0.309	−0.337
	**p**	0.229	0.119	0.860	0.734	0.109	0.074
**AXIN1**	**r**	0.368	0.018	0.039	−0.092	−0.223	−0.053
	**p**	0.054	0.925	0.845	0.636	0.253	0.786
**TRAIL**	**r**	−0.056	−0.022	0.178	−0.014	−0.103	−0.205
	**p**	0.779	0.909	0.364	0.941	0.602	0.287
**IL-20RA**	**r**	0.045	−0.279	0.122	0.164	−0.227	−0.067
	**p**	0.819	0.142	0.536	0.397	0.246	0.731
**CXCL9**	**r**	0.149	0.158	0.102	0.088	−0.411 *	−0.058
	**p**	0.449	0.414	0.607	0.649	**0.030**	0.765
**CST5**	**r**	0.380 *	−0.019	0.485 **	−0.006	0.130	0.035
	**p**	**0.046**	0.923	**0.009**	0.976	0.509	0.856
**OSM**	**r**	0.208	0.154	0.257	0.027	0.247	0.132
	**p**	0.288	0.426	0.186	0.889	0.204	0.496
**CXCL1**	**r**	−0.119	0.008	0.010	−0.265	−0.134	−0.420 *
	**p**	0.546	0.968	0.960	0.166	0.496	**0.023**
**CCL4**	**r**	0.160	0.075	0.362	0.185	−0.116	0.113
	**p**	0.415	0.698	0.058	0.337	0.558	0.560
**CD6**	**r**	0.439 *	0.175	0.447 *	0.055	−0.087	0.062
	**p**	**0.019**	0.363	**0.017**	0.778	0.661	0.751
**SCF**	**r**	−0.037	−0.091	0.125	−0.237	0.036	−0.151
	**p**	0.853	0.640	0.526	0.216	0.856	0.435
**IL-18**	**r**	0.402 *	0.433 *	0.266	0.287	−0.297	0.033
	**p**	**0.034**	**0.019**	0.172	0.131	0.125	0.865
**SLAMF1**	**r**	0.171	0.200	0.314	0.118	0.020	0.398 *
	**p**	0.384	0.299	0.103	0.541	0.921	**0.032**
**TGF-alpha**	**r**	0.088	0.063	0.101	0.017	0.121	0.079
	**p**	0.657	0.745	0.610	0.929	0.539	0.683
**MCP-4**	**r**	0.127	0.068	0.373	0.099	−0.211	−0.019
	**p**	0.520	0.726	0.050	0.609	0.280	0.920
**CCL11**	**r**	0.199	−0.040	0.301	−0.050	−0.130	0.011
	**p**	0.309	0.837	0.120	0.796	0.510	0.957
**TNFSF14**	**r**	0.172	0.015	0.078	0.008	0.036	0.075
	**p**	0.382	0.939	0.694	0.968	0.855	0.700
**MMP-1**	**r**	−0.007	−0.116	−0.095	−0.057	−0.363	−0.258
	**p**	0.971	0.548	0.630	0.770	0.058	0.176
**LIF-R**	**r**	−0.015	0.321	0.365	0.289	−0.128	−0.005
	**p**	0.938	0.090	0.056	0.128	0.515	0.978
**FGF-21**	**r**	−0.244	−0.135	0.027	−0.219	0.060	0.084
	**p**	0.211	0.485	0.890	0.253	0.764	0.665
**CCL19**	**r**	0.188	0.436 *	0.388 *	0.301	0.001	0.131
	**p**	0.339	**0.018**	**0.041**	0.112	0.997	0.499
**IL-10RB**	**r**	0.211	0.458 *	0.606 **	0.500 **	0.103	0.188
	**p**	0.282	**0.012**	**0.001**	**0.006**	0.601	0.328
**IL-18R1**	**r**	0.377 *	0.385 *	0.509 **	0.398 *	0.191	0.252
	**p**	**0.048**	**0.039**	**0.006**	**0.032**	0.331	0.186
**PD-L1**	**r**	0.240	0.159	0.276	0.322	−0.282	0.000
	**p**	0.219	0.411	0.154	0.089	0.146	0.999
**CXCL5**	**r**	−0.090	0.111	0.022	−0.079	−0.075	−0.334
	**p**	0.651	0.565	0.912	0.684	0.706	0.076
**TRANCE**	**r**	−0.128	−0.203	0.227	−0.062	−0.068	0.020
	**p**	0.517	0.290	0.246	0.751	0.732	0.917
**HGF**	**r**	0.146	0.186	0.291	0.033	0.119	0.142
	**p**	0.457	0.333	0.134	0.867	0.547	0.461
**IL-12B**	**r**	0.137	0.029	0.514 **	0.268	−0.066	0.019
	**p**	0.488	0.883	**0.005**	0.159	0.740	0.920
**MMP-10**	**r**	−0.077	0.323	0.276	0.204	−0.114	0.177
	**p**	0.697	0.088	0.155	0.289	0.565	0.357
**IL-10**	**r**	0.105	0.309	0.445 *	0.485 **	0.070	0.244
	**p**	0.594	0.103	**0.018**	**0.008**	0.725	0.201
**TNF**	**r**	0.255	0.193	0.390 *	0.207	−0.230	−0.078
	**p**	0.191	0.316	**0.040**	0.282	0.239	0.689
**CCL23**	**r**	−0.135	−0.137	0.017	−0.145	−0.447 *	−0.281
	**p**	0.493	0.479	0.932	0.452	**0.017**	0.139
**CD5**	**r**	0.083	0.210	0.348	−0.011	−0.183	−0.027
	**p**	0.673	0.273	0.070	0.953	0.351	0.889
**CCL3**	**r**	0.162	0.184	0.530 **	0.180	−0.064	0.032
	**p**	0.409	0.340	**0.004**	0.349	0.747	0.868
**Flt3L**	**r**	0.199	0.322	0.562 **	0.319	0.307	0.255
	**p**	0.311	0.088	**0.002**	0.092	0.112	0.182
**CXCL6**	**r**	0.015	0.019	0.111	0.011	−0.135	−0.246
	**p**	0.938	0.923	0.575	0.956	0.494	0.199
**CXCL10**	**r**	0.125	0.312	0.195	0.156	−0.273	−0.032
	**p**	0.525	0.100	0.320	0.420	0.161	0.869
**4E-BP1**	**r**	0.232	−0.192	0.033	−0.249	−0.158	−0.319
	**p**	0.235	0.319	0.866	0.192	0.423	0.091
**SIRT2**	**r**	0.336	0.014	−0.015	−0.140	−0.154	−0.278
	**p**	0.081	0.941	0.938	0.469	0.434	0.145
**DNER**	**r**	0.284	0.104	0.527 **	0.182	0.069	0.014
	**p**	0.143	0.590	**0.004**	0.345	0.728	0.941
**EN-RAGE(S100A12)**	**r**	0.429 *	0.198	0.074	−0.276	−0.286	−0.367
	**p**	**0.023**	0.304	0.709	0.147	0.140	0.050
**CD40**	**r**	0.229	−0.079	0.292	0.044	−0.157	0.078
	**p**	0.241	0.683	0.131	0.819	0.424	0.686
**IFN-gamma**	**r**	0.228	0.437 *	0.242	0.231	−0.333	−0.157
	**p**	0.243	**0.018**	0.214	0.228	0.083	0.416
**FGF-19**	**r**	0.316	−0.110	0.340	0.130	0.114	0.131
	**p**	0.102	0.571	0.076	0.503	0.565	0.497
**LIF**	**r**	0.366	−0.036	−0.250	−0.143	−0.079	−0.205
	**p**	0.055	0.853	0.200	0.460	0.691	0.287
**MCP-2**	**r**	0.208	0.096	0.394 *	0.095	−0.156	−0.102
	**p**	0.289	0.620	**0.038**	0.626	0.427	0.599
**CASP8**	**r**	0.451 *	−0.028	0.108	−0.194	−0.193	−0.287
	**p**	**0.016**	0.887	0.586	0.313	0.324	0.131
**CCL25**	**r**	0.231	0.077	0.632 **	0.153	0.147	0.325
	**p**	0.237	0.690	**0.000**	0.429	0.456	0.085
**CX3CL1**	**r**	0.022	−0.003	0.279	−0.008	−0.260	−0.179
	**p**	0.910	0.986	0.150	0.966	0.181	0.353
**TNFRSF9**	**r**	0.210	−0.035	0.312	−0.135	−0.178	−0.300
	**p**	0.283	0.855	0.106	0.485	0.364	0.114
**TWEAK**	**r**	0.070	−0.021	0.141	−0.069	−0.322	−0.307
	**p**	0.725	0.913	0.475	0.722	0.095	0.105
**CCL20**	**r**	0.165	0.249	0.234	−0.208	0.075	0.057
	**p**	0.403	0.193	0.230	0.278	0.706	0.767
**ST1A1**	**r**	−0.064	−0.137	−0.045	−0.145	−0.110	0.196
	**p**	0.746	0.479	0.821	0.454	0.578	0.309
**STAMBP**	**r**	0.348	−0.058	0.004	−0.219	−0.117	−0.312
	**p**	0.069	0.766	0.985	0.253	0.553	0.100
**ADA**	**r**	0.285	0.118	0.201	0.209	−0.113	−0.034
	**p**	0.142	0.543	0.304	0.277	0.568	0.862
**TNFB**	**r**	0.146	−0.023	0.083	−0.143	−0.462 *	−0.397 *
	**p**	0.459	0.905	0.674	0.460	**0.013**	**0.033**
**CSF-1**	**r**	0.205	0.103	0.104	−0.096	−0.328	−0.159
	**p**	0.296	0.595	0.599	0.620	0.088	0.410

Univariate correlations (*r*: correlation coefficient; p: *p*-value) of inflammatory serum markers and BMI, TAG and PASI. Significant correlation are highlighted in bold (*p < 0.05, ** *p* < 0.01). Non-parametric Spearman’s rank correlation method was used to assess univariate relationships between indicated markers. BMI: body mass index, TAG: triacylglycerol, PASI: psoriasis activity severity score, IL: interleukin, VEGF: vascular endothelial cell growth factor, CD: cluster of differentiation, MCP: macrophage chemotactic protein, GDNF: glial cell line-derived neurotrophic factor, CDCP1: CUB domain-containing protein 1 (CD318), OPG: osteoprotegerin, LAP: latency-associated peptide, TGF: transforming growth factor, uPA: urokinase plasminogen activator, CXCL: chemokine (C-X-C motif) ligand, TNF: tumor necrosis factor, TRAIL: TNF-related apoptosis-inducing ligand, CST: cystatin-D, OSM: oncostatin M, CCL: chemokine (C-C motif) ligand, SCF: stromal cell factor, SLAM: signaling lymphocytic activation molecule, TNFSF: TNF super family, MMP: matrix metalloproteinase, LIF-R: leukemia inhibitory factor receptor, PD-L: programmed death-ligand, TRANCE: TNF-related activation-induced cytokine, HGF: hepatocyte growth factor, Flt3: fms-like tyrosine kinase, 4E-BP: eukaryotic translation initiation factor 4E-binding protein, IFN: interferon, SIRT: NAD-dependent deacetylase sirtuin, DNER: Delta and Notch-like epidermal growth factor-related receptor, RAGE: advanced glycosylation end-product specific receptor, FGF: fibroblast growth factor, CASP: caspase, TWAEK: TNF-related weak inducer of apoptosis, STAMBP: signal-transducing adapter molecule-binding protein, ADA: adenosine deaminase, CSF: colony-stimulating factor.

**Table 2:** Multivariate linear regression analysis of inflammatory serum markers and disease activity.

**w4**	**b-Value**	***p*-Value**
lgIL-18	0.285	0.107
lgCST5	0.319	0.078
lgIL6	0.397	**0.03**
lg IL-18R	0.299	0.104
lg SIRT2	0.444	**0.019**
lgENRAGE	0.457	**0.013**
lgCASP8	0.452	**0.017**
**w8**	**b-value**	***p*-value**
lgIL-18	0.415	**0.028**
lgIL-18R	0.329	0.099
lgIL-7	0.432	**0.03**
lgIL-6	0.621	**0.001**
lgLAPTGFb	0.255	0.186
lgCCL19	0.236	0.237
lgIL-10RB	0.355	0.057
lgINFγ	0.522	**0.01**

Multivariate regression analysis calculated for reduction in indicated biomarkers (dependent variable) and reduction in disease activity (PASI) adjusted for BMI and TAG. Non-normally distributed variables as assessed by Shapiro–Wilk test were logarithmically transformed prior to multivariate testing (lg). Standardized β-coefficients and *p*-values are given. Significant correlation are highlighted in bold.

## Data Availability

Not applicable.

## Supplementary Materials

The following supporting information can be downloaded at: https://www.mdpi.com/article/10.3390/nu15071698/s1. Reference [38] is cited in the Supplementary Materials.
