# Supplementary material for: Modulation of Dietary Fatty Acids in an Open-Label Study Improves Psoriasis and Dampens the Inflammatory Activation Status
Source: Nutrients. 2023 Mar 30;15(7):1698. doi: 10.3390/nu15071698 (PMC10097201; doi:10.3390/nu15071698)
Supplement: Supplementary file 1 [file nutrients-15-01698-s001.zip › nutrients-2305225-supplementary.pdf]

Supplements

Supplement Table S1: Baseline characteristics of the study population

Patient	sex	age	age at first diagnosis	BMI	Smoker	sport	alcohol	family history	TAG	PASI	Pso-Arthritis	UV-therapy	System therapy	topical treatment
2	m	26	26	30.1	1	0	0	.	1.18	5.9	0	0	no	3
4	f	54	48	34.8	0	1	1	0	nd	3.6	0	2	no	3
6	m	37	16	25.7	2	1	1	0	nd	3	0	2	no	3
7	m	60	47	28.5	1	1	0	1	1.81	5.8	0	2	Ustekinumab	3
9	m	38	30	52	1	0	0	1	8.59	6.6	0	0	no	3
10	f	47	27	28.6	0	1	0	0	1.16	4	0	0	no	3
12	f	27	18	39.8	0	1	0	0	1.21	15.2	0	2	no	0
13	f	58	22	37.7	0	0	0	0	1.68	9.6	0	2	no	3
14	f	67	24	27.4	1	1	0	0	1.38	4.3	0	2	no	3
15	m	45	37	39.6	0	1	0	0	2.82	0.8	0	2	Fumaric acid	0
16	f	55	36	35.1	0	1	1	0	1.59	4.6	0	0	no	0
18	f	37	17	37.6	2	1	1	1	2.49	15.5	0	2	no	3
21	f	32	17	28.9	1	1	1	1	6.52	3.9	0	0	no	3
22	m	36	32	38	0	1	1	0	1.19	5.3	0	0	no	3
24	f	55	23	31.7	0	1	1	0	4.17	3.5	0	2	no	3
26	f	36	29	28.2	1	1	1	1	2.27	7.5	1	2	no	3
27	f	54	14	35.1	2	0	1	1	0.75	5.2	1	2	Guselkumab	3
28	m	71	6	36.5	0	1	0	1	2.89	9.3	0	2	MTX	1
29	m	60	4	31.6	2	1	0	1	2.74	4.2	1	2	no	3
31	m	52	51	28.8	2	1	1	0	0.65	3.8	0	0	no	3
33	f	39	15	36.1	1	0	1	0	1.44	9.2	0	2	no	3
34	f	44	41	35.7	1	1	1	1	1.69	6.9	0	0	MTX	2
36	f	67	61	40.3	2	0	1	1	4.24	6	0	0	no	2
37	f	38	25	29.7	1	0	1	1	1.79	3	0	0	no	2
39	f	70	23	32.3	0	0	0	0	3.26	9.5	0	0	Fumaric acid	3
41	m	61	52	38.1	1	0	1	0	3.2	2.9	0	0	no	2
42	m	31	19	29.8	0	1	1	1	0.86	2.4	1	2	Apremilast	2
43	f	32	26	42.5	0	0	0	0	1.41	14.4	0	0	MTX	3
44	f	48	37	37.2	2	0	0	0	2.29	5.2	0	2	no	3
45	m	62	54	38.3	0	0	0	1	2.41	4	0	0	no	2
46	m	23	6	30.8	0	1	1	1	1.13	5.6	0	2	no	3
47	f	41	12	35.2	1	0	1	1	1.99	6	0	2	no	3
48	f	40	12	31.7	0	1	1	0	0.96	3.6	0	0	no	2

BMI: body mass index; TAG: triglycerides; MTX: methotrexate, Sex (m=male; f= female), age in years, smoker (0=no, 1=yes, 2=ex-smoker), sport, alcohol, family history, psoriasis arthritis, (0=no, 1=yes), UV-therapy (0=no, 2 only in the past i.e. terminated > 2 months before study inclusion), Topical treatment (0=no, 1=Calcipotriol, 2=Glucocorticoid, 3= Calcipotriol and Glucocorticoid), nd: not detected

Supplemental Table S2: Diet composition and application

**Formula diet**	**Per 100 g / per meal (dissolved in water)**
carbohydrate	29.1 g
protein	52.2 g
fat	1.8 g
Saturated fatty acids	0.5 g

Plan of diet from week 1 to week 12

<table>
<tr><th>week</th><th>formula diet</th><th>meal according to the Mediterranean diet</th><th>drinks</th><th>in case of hunger</th></tr>
<tr><td>week 1</td><td>3 times / day</td><td>-</td><td rowspan="3">free of sugar and sweetener</td><td rowspan="3">vegetables or wheat bran</td></tr>
<tr><td>week 2 and 3</td><td>2 times / day</td><td>1 time / day</td></tr>
<tr><td>week 4 - 12</td><td>1 time / day</td><td>2 times / day</td></tr>
</table>

Preparation of formula diet

formula diet (Almased^{TM})	100 g
either: water<br>or low-fat milk<br>or buttermilk<br>or sugar-free tea	300 ml<br>200 ml<br>200 ml<br>200 ml
vegetable oil (canola oil/soybean oil/linseed oil/walnut oil)	2 teaspoons (6 g)
cinnamon, turmeric or baking cocoa	if desired

Diet recommendations

**food components**	**recommended intake**
side dishes (bulgur, potatoes, whole-grain pasta, couscous, wholemeal bread, oatmeal)	4 portions / day
vegetables	at least 3 portions / day
fruit	not more than 2 portions /day

extra virgin olive oil, olives	daily
nuts	daily
legumes	daily
milk and dairy products (yogurt and cheese)	4 portions / week
eggs	3 portions / week
fish	2 – 3 portions / week
poultry	2 portions / week
red meat	1 portion / month
sweets/savory snacks	not more than 2 portions / week

Individual patient care

At the start of the study, the patients kept a food diary for 7 days which was analyzed by us. Macro- and micronutrients were calculated and the intake of omega-3 fatty acids was assessed. In addition, the individual energy requirements of the patients were calculated in order to apply the correct dosage of the formula diet.

Patients received detailed counselling on the use of the formula diet, which was conducted as a complete meal replacement from day 1 to day 7. Vegetables were allowed without restriction and wheat bran was used for fiber enhancement when needed. According to the diet plan, fixed times for food intake were discussed and a break time of approximately 4 to 5 hours was scheduled between meals. Cravings were also discussed and appropriate strategies were developed with the patients. To increase taste acceptance of the formula diets, admixtures of unsweetened additives such as pureed vegetables and spices were discussed.

Each of the following days was evaluated individually:

Day 8 and day 9: Two formula diet meals and the dinner was defined as 1 to 2 slices of whole-grain bread, margarine or low-fat cottage cheese, and lean sausage. Vegetables ad libitum and wheat bran if needed.

Day 10 to day 21: Two meals with formula diet and the dinner was defined with 1 to 2 slices of whole-grain bread, margarine or low-fat cottage cheese and cheese (max. 30% fat in dry matter) or lean sausage. Vegetables ad libitum and wheat bran if needed.

From day 22: One meal with formula diet, the other meals defined following the Mediterranean diet with omega-3 fatty acid-rich foods. Additional cooking recipes were provided.

For each nutritional visit, a nutritional protocol was evaluated as a 24-hour recall with subsequent evaluation and nutritional counseling.

The dietary recommendations corresponded in each case to the 10 rules of the German Nutrition Society below and the use of omega-3 fatty acid-rich foods (walnuts, flaxseeds, chia seeds, algae, linseed oil, rapeseed oil, soybean oil, walnut oil). The food pyramid of the anti-inflammatory diet, described by Rondanelli et al., has not been used directly. However, it corresponds in many parts to our recommendations [1]. However, portion sizes were not communicated as strictly. Wine was not recommended separately.

10 guidelines of the German Nutrition Society (DGE) for a wholesome diet

A wholesome diet keeps you in good health and promotes vitality and well-being. The 10 dietary guidelines of the DGE summarize how a wholesome diet can be implemented and they are based on recent scientific knowledge.

1. Enjoy food diversity
2. Vegetables and fruit – take “5 a day”
3. Favor whole-grain foods
4. Complete the choice with animal-based foods
5. Choose health-promoting fats
6. Reduce sugar and salt intake
7. Water is the best choice
8. Prepare carefully cooked dishes
9. Mindful eating and enjoying is important.
10. Watch your weight and stay active

1. Rondanelli, M.; Faliva, M.A.; Miccono, A.; Naso, M.; Nichetti, M.; Riva, A.; Guerriero, F.; De Gregori, M.; Peroni, G.; Perna, S. Food Pyramid for Subjects with Chronic Pain: Foods and Dietary Constituents as Anti-Inflammatory and Antioxidant Agents. *Nutr Res Rev* **2018**, *31*, 131–151, doi:10.1017/S0954422417000270.
